# Duration of skin-to-skin care and rectal temperatures in late preterm and term infants

**DOI:** 10.1186/s12884-022-04983-7

**Published:** 2022-08-20

**Authors:** Darjan Kardum, Edward F. Bell, Boris Filipović Grčić, Andrijana Müller

**Affiliations:** 1 Department of Pediatrics, University Hospital Osijek, J. Huttlera 4, 31000 Osijek, Croatia; 2 University of Osijek, School of Medicine, J. Huttlera 4, 31000 Osijek, Croatia; 3 Department of Pediatrics, University of Iowa, 200 Hawkins Drive, 52242 Iowa City, IA USA; 4 Department of Pediatrics, University Hospital Zagreb, Kišpatićeva 12, 10000 Zagreb, Croatia; 5 University of Zagreb, School of Medicine, Šalata 3, 10000 Zagreb, Croatia; 6 Department of Gynecology and Obstetrics, University Hospital Osijek, J. Huttlera 4, 31000 Osijek, Croatia

**Keywords:** Birth, Body temperature regulation, Skin-to-skin care, Delivery rooms, Hypothermia

## Abstract

**Background:**

Hypothermia during the newborn period is widely regarded as a major contributory cause of significant morbidity and mortality of newborn infants. Thermoprotective behaviours such as skin-to-skin care (SSC) or the use of appropriate devices have been recommended as simple tools for the avoidance of neonatal hypothermia. We examined the relation between the duration of skin-to-skin care and infant temperature change after birth in suboptimal delivery room temperatures.

**Methods:**

We reviewed the medical charts of all vaginally born infants of gestational age ≥ 35 weeks born January-July 2018 and admitted to the well-baby nursery. After SSC was discontinued, the infant’s rectal temperature was measured to determine the frequency and severity of hypothermia.

**Results:**

The charts of 688 vaginally born infants were examined. Our mean delivery room temperature was 21.7 (SD 2.2) °C, well below the WHO recommendation of 25 °C. After SSC 347 (50.4%) infants were normothermic (temperature 36.5–37.5 °C), 262 (38.0%) were mildly hypothermic (36.0-36.4 °C), and 79 (11.4%) were moderately hypothermic (32.0-35.9 °C). The mean skin-to-skin time in infants was 63.9 (SD 20.9) minutes. SSC duration was associated with increase in rectal temperature for patients of gestational ages ≥ 38 weeks and with decrease in rectal temperature in patients of gestational age < 38 weeks.

**Conclusion:**

SSC is effective, even at suboptimal delivery room temperatures, for promoting normothermia in infants of ≥ 38 weeks’ gestation but may not provide adequate warmth for infants of < 38 weeks.

## Background

Hypothermia during the newborn period is widely regarded as a major contributory cause of significant morbidity and mortality around the world and especially in developing countries [1]. Among infants born in the hospital, the prevalence of hypothermia, defined as temperature below 36.5 °C, is 32–85%, and in those infant born at home, it is even higher, up to 92%, even in tropical environments [2].

Thermoprotective behaviours such as skin-to-skin care or the use of appropriate devices have been recommended as simple tools for the avoidance of neonatal hypothermia [3]. Skin-to-skin care (SSC) is defined by the World Health Organization (WHO) as: “*when the infant is placed prone on the mother’s abdomen or chest in direct ventral-to-ventral skin-to-skin contact. Immediate skin-to-skin contact is done immediately after delivery, less than 10 minutes after birth. Early skin-to-skin contact was defined as beginning any time from delivery to 23 hours after birth. Skin-to-skin contact should be uninterrupted for at least 60 minutes*” [4].

The WHO proposes a “warm chain,“ a set of 10 interlinked procedures carried out at birth and during the following hours and days [4]. To be implemented in institutions and (in an abridged form) at home, the “warm chain” aims to minimize the risk of hypothermia in newborns with measures aimed at achieving adequate delivery room temperatures, immediate drying of the skin, skin-to-skin care (SSC), early and exclusive breastfeeding, delaying bathing, appropriate clothing and bedding, promoting mother and infant contact, and in institutions, practices that emphasize warm transportation, warm resuscitation, and training to raise awareness of this important issue [4].

Even though skin-to-skin care is simple and has numerous benefits besides thermal control [5], its full application remains challenging due to several factors. The major barriers identified are lack of personnel (nurses), time constraint, difficulty in deciding on eligibility for SSC, safety concerns, interference with clinical routines, and interdepartmental issues. Recall of an adverse event during SSC was also a major barrier [6].

Globally, the prevalence of skin-to-skin care varies widely [7] from 8% reported in Ethiopia [8] to 92% in Australia [9].

In this study, we evaluated how the duration of skin-to-skin care impacts the change in temperature until transfer to well-baby nursery in healthy newborns. Similar studies have shown that skin-to-skin care was at least as effective as incubator care for rewarming hypothermic low-risk neonates [10]. Thus, implementation of skin-to-skin care may be an important approach, especially in countries with limited resources.

However, it is still unknown how the duration of skin-to-skin contact affects temperature increase or decrease in late preterm infants and early term or full-term neonates. Also, it is unknown whether lower than recommended delivery room temperatures influence net gain or loss of heat during the SSC period.

## Methods

### Study design and population

This retrospective cohort study was performed in the delivery rooms and well-baby nursery at a regional University Hospital in Osijek, Croatia. We reviewed the medical charts of all vaginally born infants of gestational age ≥ 35 weeks born from January 2018 to July 2018 and admitted to the well-baby nursery. All infants born vaginally received skin-to-skin care, defined as placing the newborn prone on the mother’s abdomen or chest in direct ventral-to-ventral skin-to-skin contact, drying the infant and covering it with a preheated blanket and placing a cotton cap on the infant’s head. The study was approved by the University Hospital Osijek Institutional Ethics Committee.

### Measurements

The duration of skin-to-skin care was determined by the mother and was recorded. After skin-to-skin care was discontinued, the nurse measured the infant’s temperature rectally (using a Gima S.p.A. Digital Thermometer) and recorded on the medical chart. The time of temperature measurement is noted on the chart. Ambient temperatures in the delivery room were measured using a multi-purpose thermometer (SD-Duvančić) every three days at 7 AM, 3 PM, 11 PM, for the observed period.

On admission to the well-baby nursery, the infants were evaluated and treated for hypothermia, if present, using the EQUATOR® Convective Warming System or a preheated incubator. Admission hypothermia was defined according to the World Health Organization as rectal temperature < 36.5 °C, with the following subclassification: mild hypothermia or cold stress, 36.0 to 36.4 °C; moderate hypothermia, 32.0 to 35.9 °C; and severe hypothermia below 32.0 °C [4].

### Statistical analysis

Categorical data are represented by absolute and relative frequencies. Numerical data were described by the arithmetic mean and standard deviation in the case of normal distributions and, in the other cases, by the median and interquartile range (IQR). Differences of categorical variables were tested by Chi-square and Fisher’s exact tests. The normality of the distribution of numerical variables was tested by the Shapiro-Wilk test. Differences in normally distributed numerical variables between two independent groups were tested by Student’s t-test and, in non-normally distributed variables, by Mann-Whitney’s U test. The correlation between numerical variables was evaluated using Spearman’s correlation coefficient ρ. All *P* values were two-sided; the significance level was set at alpha 0.05 [11]. The statistical analysis was performed using MedCalc Statistical Software version 19.4.1 (MedCalc Software Ltd, Ostend, Belgium; https://www.medcalc.org; 2020), and SPSS 17.0 (SPSS Inc., Chicago, IL, USA).

## Results

The charts of 688 vaginally-born infants were examined. The characteristics of these patients are shown here (Table 1).

**Table 1:** Patient characteristics and outcomes in vaginally born infants (*n* = 688)

Characteristics	Value
Gestational age (completed weeks) [mean (SD)]	39.0 (1.1)
Birth weight (g) [mean (SD)]	3401 (453)
Female [n (%)]	344 (50.0)
SGA [n (%)]	48 (7.0)
LGA [n (%)]	28 (4.1)
Time of temperature measurment, minutes [mean (SD)]	63.9 (20.9)
Admission temperature, °C [mean (SD)]	36.4 (0.5)
Hypoglycemia on admission [n (%)]	18 (2.6)
Active warming^a^ [n (%)]	105 (15.3)
Oxygen supplementation [n (%)]	50 (7.3)
Antibiotic treatment [n (%)]	35 (5.1)
Mild hypothermia [n (%)]	262 (38.1)
Moderate hypothermia [n (%)]	79 (11.5)

^a^Using the convective warming system or a preheated incubator

After SSC 347 (50.4%) infants were normothermic, 262 (38.0%) were mildly hypothermic and 79 (11.4%) were moderately hypothermic. There were no severely hypothermic infants and no hyperthermic patients. The mean SSC time in infants born vaginally was 63.9 (SD 20.9) minutes. The mean air temperature in the delivery room during the study period was 21.7 (SD 2.2) °C.

Compared to normothermic infants, infants with moderate hypothermia were shorter gestational age (38.7 vs. 39.1 weeks) and lower birth weight (3088.1 vs. 3514.2 g) and were more often small for gestational age (21.5% vs. 4.3%). Regarding short-term interventions, they more often required oxygen supplementation and active warming by radiant heat or icubator (Table 2).

**Table 2:** Characteristics and short-term outcomes among normothermic, mildy hypothermic and moderately hypothermic vaginally born infants

	Normothermia (*n* = 347)	Mild hypothermia (*n* = 262)	P^a^ (normothermia vs. mild hypothermia)	Moderate hypothermia (*n* = 79)	P^a^ (normothermia vs. moderate hypothermia)
Gestational age (completed weeks) [mean (SD)]	39.1 (1.1)	38.9 (1.2)	**0.03**^b^	38.7 (1.2)	**0.004**^b^
Birth weight (g), [mean (SD)]	3514 (438)	3345 (428)	**< 0.001**^b^	3088 (422)	**< 0.001**^b^
Female [n (%)]	167 (48.1)	133 (50.8)	0.57	44 (55.7)	0.26
SGA [n (%)]	15 (4.3)	16 (6.1)	0.35	17 (21.5)	**< 0.001**
LGA [n (%)]	19 (5.5)	9 (3.4)	0.25	0	**0.03**
Maternal fever [n (%)]	7 (2.0)	4 (1.5)	0.76	0	0.36
Time of temperature measurment, minutes [mean (SD)]	65.6 (21.9)	61.5 (18.2)	**0.01**^b^	64.5 (23.9)	0.69^b^
Rectal temperature, °C [mean (SD)]	36.8 (0.3)	36.2 (0.2)	**< 0.001**^b^	35.6 (0.3)	**< 0.001**^b^
Hypoglycemia on admission [n (%)]	10 (2.9)	5 (1.9)	0.60	3 (3.8)	0.72
Active warming^d^ [n (%)]	26 (8.0)	27 (10.3)	0.25	52 (65.8)	**< 0.001**
Oxygen supplementation [n (%)]	23 (6.6)	15 (5.7)	0.74	12 (15.2)	**0.02**
Antibiotic treatment [n (%)]	16 (4.6)	12 (4.6)	> 0.99	7 (8.9)	0.16
Length of stay, days [median (IQR)]	3 (3–4)	3 (3–4)	> 0.99^‡^	3 (3–5)	0.09^c^

^a^Fisher’s Exact test;^b^Student t-test; ^c^Mann Whitney U test; ^d^using the convective warming system or a preheated incubator

There was a positive correlation between the duration of skin-to-skin care and the increase in rectal temperature; however, this relationship was not statistically significant (ρ 0.054, *P* 0.39).

SSC duration was positively correlated to increase in recorded temperatures in infants of gestational age ≥ 38 weeks and negatively in patients of gestational age < 38 weeks. The relation of admission temperature to the duration of SSC is shown for each gestational week in Table 3.

**Table 3:** Relation of rectal temperatures to skin-to-skin duration by gestational age

Gestational age (completed weeks)	Spearman’s rank correlation coefficient of time and measured temperature ρ (*P* value)	Trend equation
≤ 36 (*n* = 29)	− 0.138 (0.58)	y = − 0.004 x minutes + 36.67 °C
37 (*n* = 68)	− 0.087 (0.54)	y = − 0.002 x minutes + 36.36 °C
38 (*n* = 203)	0.138 (0.12)	y = 0.003 x minutes + 36.40 °C
39 (*n* = 335)	0.169 (0.008)	y = 0.004 x minutes + 36.23 °C
40 (*n* = 234)	0.097 (0.20)	y = 0.002 x minutes + 36.49 °C
≥ 41 (*n* = 70)	0.248 (0.06)	y = 0.002 x minutes + 36.63 °C

## Discussion

Our study aimed to determine whether SSC is effective at maintaining normothermia in suboptimal delivery room temperatures. Delivery room air temperatures averaged 21.7 (SD 2.2) °C during the observed period. The ideal therapeutic goal of thermal care is to keep the newborn in the zone of thermal neutrality, the environmental temperature range in which the body temperature is normal and oxygen consumption is minimal [12]. One of the 10 interlinked procedures that the WHO recommends [4] to be carried out at birth and during the following hours and days is the warming the delivery place in preparation for birth to at least 25 °C and keeping the birthplace free from draughts [4].

The simple intervention of warming the delivery place is most often practically challenging even in well-developed countries due to a variety of reasons, i.e. for the comfort of the labouring mother and staff including surgical personnel who must wear multiple layers of protective clothing [6, 13].

Several studies have shown that SSC is effective in achieving and maintaining normothermia in term neonates. A meta-analysis [14] showed that SSC leads to higher breastfeeding rates and duration and improved physiological stability. Regarding infant thermoregulation, five of the six studies included in the meta-analysis found that axillary temperatures were higher in SSC infants. However, of the studies included in the meta-analysis, two were performed with delivery room temperatures higher than those recommended by the WHO [15, 16], and the other four studies did not report room temperatures where SSC was carried out [5, 17–19].

As was recently shown, increasing delivery room temperatures to WHO recommended temperatures was effective in reducing cold stress even in very premature neonates [13]. A study from Germany showed almost complete elimination of admission hypothermia in very low birth weight (VLBW) neonates when delivery room temperatures were raised from 28 to 34 °C but also found an increase in hyperthermia (body temperature > 37.5 °C) in preterm babies [20].

In our study, out of 688 infants receiving SSC, 347 (50.4%) infants were normothermic, 262 (38.0%) were mildly hypothermic, and 79 (11.4%) were moderately hypothermic when the SSC was discontinued. These rates of hypothermia are similar to those reported by Lunze et al. [2].

Compared to normothermic infants, infants with moderate hypothermia were shorter gestational age (38.7 vs. 39.1 weeks), lower birth weight (3088 vs. 3514 g) and were more often small for gestational age (21.5% vs. 4.3%). They more often required oxygen supplementation (Table 2). This finding is consistent with the proposed physiology of temperature regulation in infants since oxygen consumption of the human neonate increases two to three fold during cold stress at birth [21].

SSC duration was overall positively correlated to an increase in rectal temperatures even at suboptimal delivery room temperatures (Table 3). However, when evaluating the relationships among duration of SSC with rectal temperatures and gestational age, in infants < 38 weeks gestation, we observed a decreasing trend in rectal temperatures after SSC. Lower gestational age is a known risk factor for neonatal hypothermia in preterm infants of GA ≤ 30 gestational weeks [22]. Our study confirms this finding even in infants < 38 weeks gestation compared to infants ≥ 38 weeks gestation in suboptimal delivery room temperatures. In this environment, prolonged SSC could lead to higher rates of neonatal hypothermia in these infants with diminished adaptive mechanisms due to lower gestational age.

There are several limitations to our study. Delivery room temperatures were recorded every three days at 7 AM, 3 PM, 11 PM, for the observed period and not recorded at the time of each birth. Also, the maternal temperature was not recorded at the time of birth and it affects the temperature of the newborn. It is known from previous studies that the temperature of the fetus is 0.3–0.5 °C higher than that of the mother [21]. Furthermore, the duration of SSC varied since it was determined by the mother, and this could have impacted the results. For example mothers of more immature infants could have SSC discontinued before positive effects of SSC started, and this could impact overall findings of the study.

## Conclusion

Our study found that SSC is effective for promoting normothermia in infants of gestational age ≥ 38 weeks, even at suboptimal delivery room temperature. However, higher delivery room temperatures, as recommended by WHO, are advised if SSC is to be applied at birth for preterm infants, even those who are late preterm.

## Data Availability

The datasets used and/or analysed during the current study are available from the corresponding author on reasonable request.

## Abbreviations

SSC: Skin-to-skin care
WHO: World Health Organization
IQR: Interquartile range
SD: Standard deviation
